# Quantitative iTRAQ-based proteomic analysis of differentially expressed proteins in aging in human and monkey

**DOI:** 10.1186/s12864-019-6089-z

**Published:** 2019-10-11

**Authors:** Hao Wang, Xiaoqi Zhu, Junyan Shen, En-Feng Zhao, Dajun He, Haitao Shen, Hailiang Liu, Yongxin Zhou

**Affiliations:** 1 Department of Thoracic-Cardiovascular Surgery, Tongji Hospital, Tongji University School of Medicine, Shanghai, China; 2 Translational Center for Stem Cell Research, Tongji Hospital, Tongji University School of Medicine, Shanghai, 200065 China; 3 College of Life Sciences, Key Laboratary of Xinjiang Phytomedicine Resource and Utilization of Ministry of Education, Shihezi University, Shihezi, 832003 Xinjiang China

**Keywords:** Plasma, Quantitative proteomics, iTRAQ, IGFBP4, Cognitive dysfunction

## Abstract

**Background:**

The underlying physiological mechanisms associated with aging are still complex and unclear. As a very important tissue of human body, the circulatory system also plays a very important role in the process of aging. In this study, we use the isobaric tags for relative and absolute quantification (iTRAQ) method to identify differentially expressed proteins in plasma for humans and monkeys between young and aged. Western blotting and behavioral experiment in mice were performed to validate the expression of the candidate protein.

**Results:**

Between the young / the old humans and the young / the old monkeys 74 and 69 proteins were found to be differently expressed, respectively. For the human samples, these included 38 up-regulated proteins and 36 down-regulated proteins (a fold change ≥1.3 or ≤ 0.667, *p* value ≤0.05).For the monkey samples, 51 up-regulated proteins and 18 down-regulated proteins (a fold change ≥1.3 or ≤ 0.667, *p* value ≤0.05). KEGG pathway analysis revealed that phagosome, focal adhesion, ECM-receptor interaction and PI3K/AKT signaling pathway were the most common pathways involved in aging. We found only IGFBP4 protein that existed in up-regulated proteins in aged both for human and monkey. In addition, the differential expression of IGFBP4 was validated by western blot analysis and IGFBP4 treatment mimicked aging-related cognitive dysfunction in mice.

**Conclusions:**

This first, the integrated proteomics for the plasma protein of human and monkey reveal one protein-IGFBP4, which was validated by western blotting and behavioral analysis can promote the process of aging. And, iTRAQ analysis showed that proteolytic systems, and inflammatory responses plays an important role in the process of aging. These findings provide a basis for better understanding of the underlying mechanisms involved in aging.

## Background

Aging, which is broadly defined as the time-dependent functional decline that affects most living organisms, has attracted curiosity and excited imagination throughout the history of humankind [1]. Nowadays, aging is known as a physiological process encompassing several body changes at macroscopic and microscopic levels throughout an organism’s lifespan. Heart, brain, and muscle are considered as the most efficient model systems or tissues with the ability to affect aging and lifespan [2–5]. Through the study of various age-related diseases, such as Alzheimer’s disease, hypertension, and Parkinson’s disease, links between these age-related diseases and these model systems have been established [6, 7]. There are several factors, such as genomic instability, telomere attrition, epigenetic alterations, loss of proteostasis, deregulated nutrient sensing, mitochondrial dysfunction, cellular senescence, stem cell exhaustion, and altered intercellular communication, involved in the progression of aging [1, 2]. Nevertheless, due to the complexity of the molecular mechanisms involved in aging, we have not yet fully explained the causes, processes, and relationships between them.

Many previous studies have revealed differences between older and younger species at both the biological and molecular levels [1]. A large mountof evidence indicates that tumor necrosis factor α (TNF-α), a Transforming growth factor (TGF)-secreted factor superfamily member, and ubiquitination and ubiquitin-like family proteins may play critical roles in the progression of aging [8–10]. Some differentially expressed genes involved in the process of aging have been revealed by microarray-based whole-genome gene expression analyses [11]. A comprehensive and in-depth study of proteomics will help us to better understand the mechanism of aging.

Tissue-based quantitative clinical proteomics has emerged as an unbiased discovery tool to study the mechanisms of various diseases [12]. Although many targeted investigations of aging have been designed to examine specific pathways that are significant to this phenomenon [13, 14], few studies have focused on the expression landscape of overall proteins in plasma, especially for human and monkey. However, plasma, an important component of the blood which has the central and integrating role in human physiology, can reflect the physical condition of an individual [15]. For mice, some systemic factors in plasma can affect cognitive function, either positively by promoting neurogenesis, or negatively by reducing neurogenesis [16–19]. Therefore, we compared the differentially expressed proteins in plasma between young and aged subjects using iTRAQ-based quantitative proteomics to gain information to understand the mechanism of aging at the protein level.

## Results

### Characteristics of the clinical study

We collected plasma samples from eight humans and eight monkeys. The human and monkey samples were equally divided into four different groups based on their age. There was a significant difference in age between the young and the aged. (*p* < 0.05). Table 1 shows the detailed information of the samples.

**Table 1:** Characteristics of aged and young samples in this study

	Human			Monkey		
	Aged	Young	*P* value	Aged	Young	*P* value
Health Status	Health	Health	/	Health	Health	/
Age (year), mean (SD)	87.25, 9.287	26.25, 2.986	< 0.0001	21.75, 4.425	7.5, 1.291	0.0008
Female	86.00, 8.485			21.5, 6.364		
		/	0.8446		/	0.9348
Male	88.50, 13.44			22.0, 4.243		

### iTRAQ quantification of plasma protein profiles

**Fig. 1:**
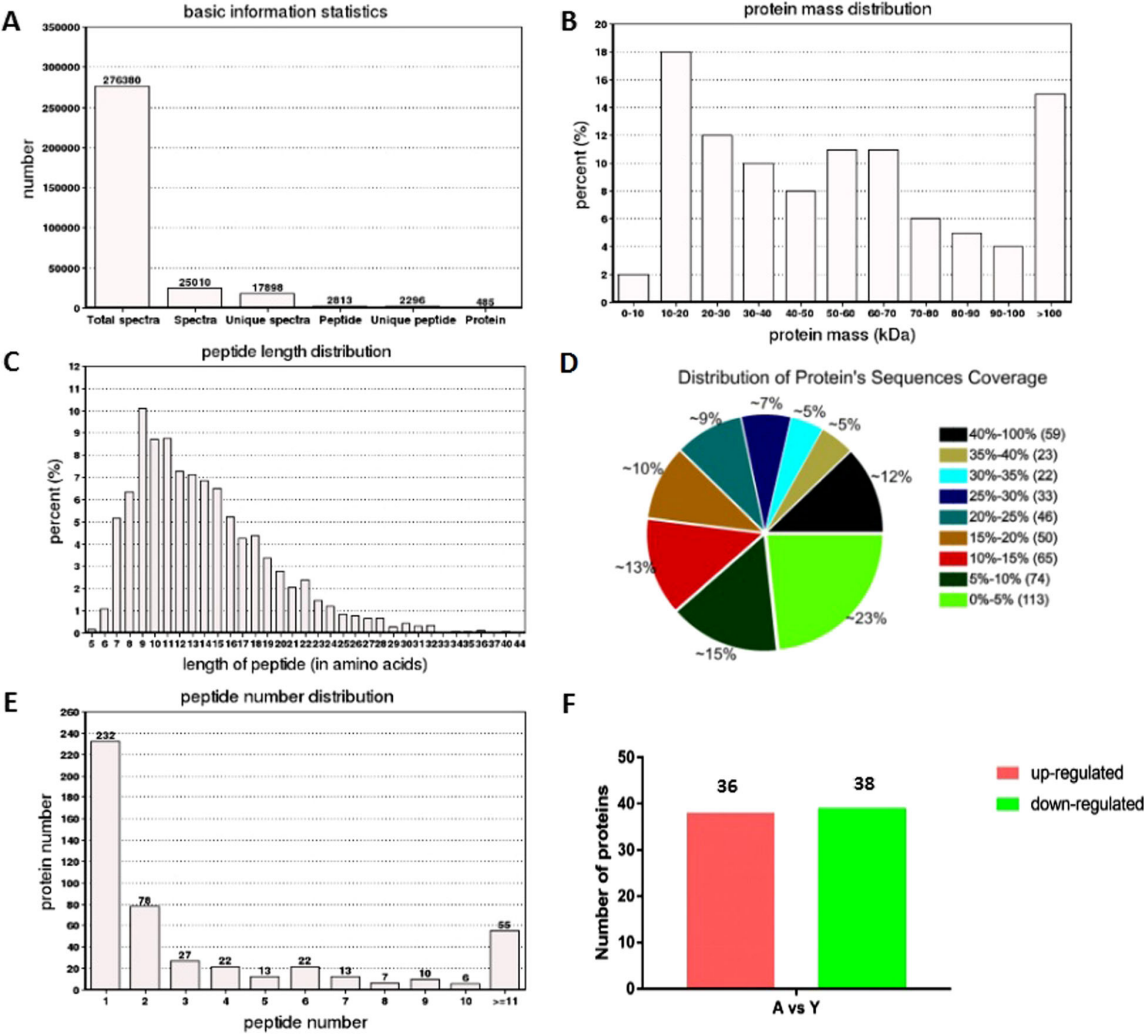
The human plasma proteome dataset from iTRAQ shotgun analysis. (**a**) A statistical chart for identifying basic information. Total spectra is the total number of secondary spectra, Spectra is the number of matched spectra, Unique Spectra is the number of matched peptides, Peptide is the number of peptides identified, Unique Peptide is the number of specific peptide sequences, and Protein is the number of proteins identified. (**b**) Mass distribution map of proteins based on molecular weight (kDa). (**c**) Distribution of different peptide length. (**d**) Distribution of peptide sequence coverage. (**e**) The quantitative distribution of identified peptides. (**f**) Quantity statistics of differential proteins

We employed iTRAQ labeling technology in combination with LC-MS/MS to investigate differentially expressed proteins in circulating blood between the aged and young groups. In total, 485 and 708 proteins were identified to be commonly expressed in human and monkey samples, respectively (Fig. 1a and Additional file 1: Figure S1A). These proteins were subjected to further analysis. Among the 485 identified human proteins, approximately 98% were more than 10 kDa (Fig. 1b). Approximately 95% of peptides were between 6 and 24 amino acid residues in length (Fig. 1c). Of the identified proteins, more than 62% had sequence coverage above 10% (Fig. 1d). More than 88% of the proteins contain less than 10 peptides, and the number of proteins decreases with the increase in the number of matching peptides (Fig. 1e).

For monkey samples, among the 708 identified proteins, approximately 98% were more than 10 kDa (Additional file 1: Figure S1B). Approximately 96% of peptides were between 6 and 24 amino acid residues in length (Additional file 1: Figure S1C). Of the identified proteins, more than 44% had sequence coverage above 10% (Additional file 1: Figure S1D). More than 90% of the proteins contain less than 10 peptides, and the number of proteins decreases with the increase in the number of matching peptides (Additional file 1: Figure S1E).

**Fig. 2:**
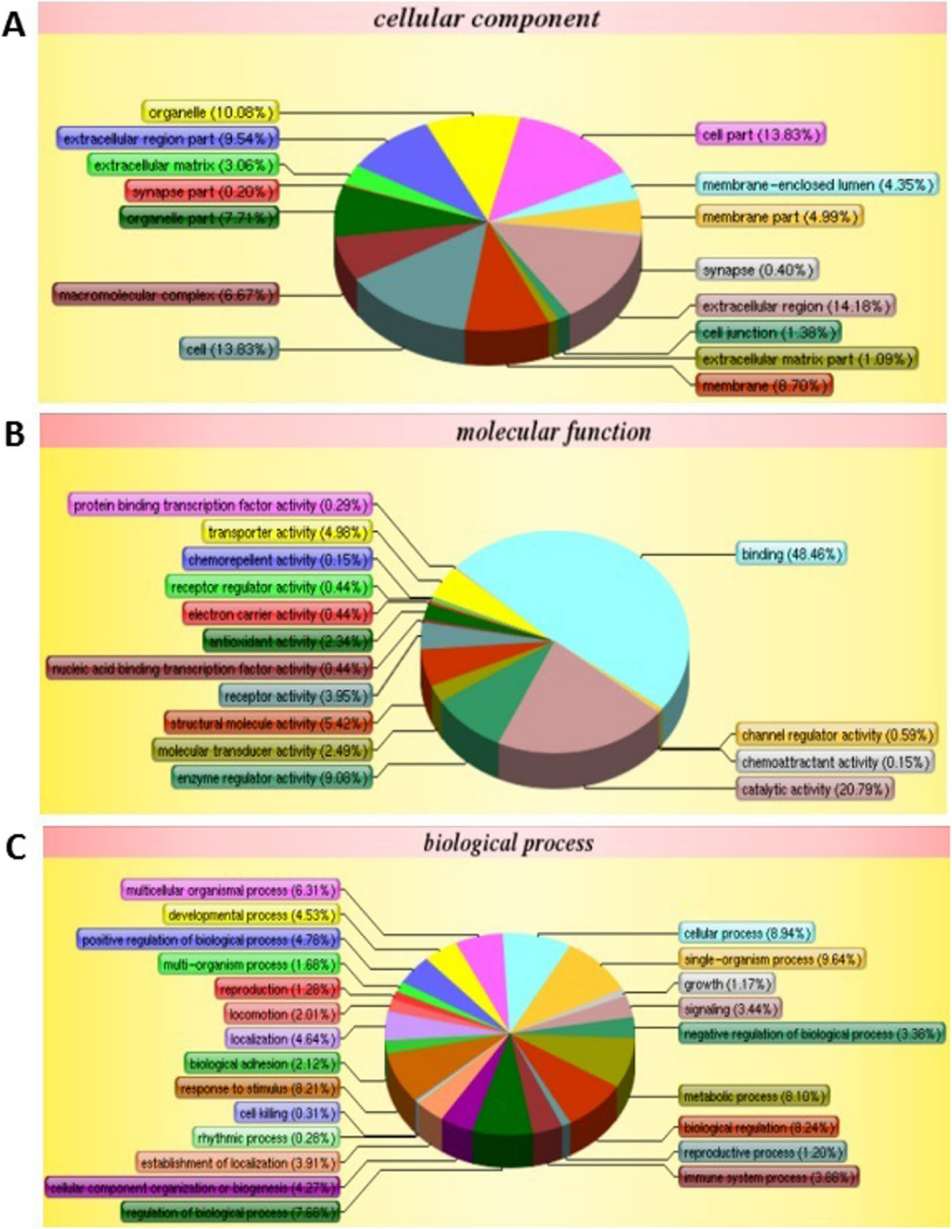
Gene ontology (GO) annotation and functional classification of identified serum proteins from all human samples. GO terms for cellular compartment (**a**), molecular function (**b**), and biological process (**c**)

The subcellular localizations, molecular functions, and biological processes of identified proteins were analyzed by GO annotation. For human samples, extracellular region (14.18%), cell part (13.83%), cell (13.83%) and organelle (10.08%) were the most representative cell component classifications among the identified proteins (Fig. 2a). In terms of molecular functions, the most representative groups of the identified proteins were binding (48.46%), catalytic activity (20.79%), and enzyme regulator activity (9.08%) (Fig. 2b). In biological processes,single-organism process (9.64%), cellular process (8.94%), biological regulation (8.24%), response to stimuli (8.21%), metabolic process (8.1%) were the most representative of the processes (Fig. 2c).

In monkey samples, cell part (15.52%), cell (15.52%), organelle (12.29%) and extracellular region (9.33%) were the most representative cell component classifications among the identified proteins (Additional file 2: Figure S2A). In terms of molecular functions, the most representative groups of the identified proteins were binding (47.72%), catalytic activity (21.16%), structural molecule activity (8.25%) and enzyme regulator activity (7.69%) (Additional file 2: Figure S2B). In biological processes, cellular process (9.71%) single-organism process (9.56%), metabolic process (8.49%), biological regulation (7.86%), regulation of biological process (7.34%) and response to stimuli (7.23%) were the most representative of the processes (Additional file 2: Figure S2C).

### Differential levels of plasma proteins between aged and young samples

For the human samples, of the 485 identified proteins, 38 were up-regulated (ratio Aged/Young ≥1.3, *p* < 0.05) and 36 were down-regulated (ratio Aged/Young ≤0.667, *p* < 0.05) (Tables 2 and 3). For the monkey samples, of the 708 identified proteins, 51 were up-regulated (ratio Aged/Young ≥1.3, *p* < 0.05) and 18 were down-regulated (ratio Aged/Young ≤0.667, *p* < 0.05) (Additional file 3: Table S1 and Additional file 4: Table S2). Although the similarity of the differentially expressed proteins in humans and monkeys was not high, we found only IGFBP4 protein that existed in up-regulated proteins in aged both for human and monkey. Then it was focused for subsequent analysis.

**Table 2:** Up-regulated proteins, identified in aged of human

Number	Accession	Protein symbol	Description	Gene symbol	Mean ± SD	E_Value
1	Q15113	PCOC1	Procollagen C-endopeptidase enhancer 1	PCOLCE	1.92 ± 0.53	0
2	P05090	APOD	Apolipoprotein D	APOD	1.58 ± 0.3	1.00E-95
3	Q9UHG3	PCYOX	Prenylcysteine oxidase 1	PCYOX	1.58 ± 0.51	0
4	P03950	ANGI	Angiogenin	ANG	1.49 ± 0.41	1.00E-73
5	O76076	WISP2	WNT1-inducible-signaling pathway protein 2	WISP2	1.89 ± 0.62	5.00E-89
6	P00450	CERU	Ceruloplasmin	CER	1.28 ± 0.34	0
7	P0CG05	LAC2	Ig lambda-2 chain C regions	IgG LC	1.34 ± 0.28	5.00E-54
8	P07988	PSPB	Pulmonary surfactant-associated protein B	SFTPB	1.44 ± 0.42	0
9	Q86UD1	OAF	Out at first protein homolog	OAF	1.47 ± 0.4	1.00E-141
10	Q9HDC9	APMAP	Adipocyte plasma membrane-associated protein	APMAP	1.7 ± 0.83	0
11	P05160	F13B	Coagulation factor XIII B chain	F13B	1.46 ± 0.5	0
12	P27918	PROP	Properdin	CFP	2.53 ± 0.27	0
13	P06727	APOA4	Apolipoprotein A-IV	APOA	1.64 ± 0.36	0
14	P02745	C1QA	Complement C1q subcomponent subunit A	C1QA	1.23 ± 0.24	7.00E-126
15	P22692	IBP4	Insulin-like growth factor-binding protein 4	IGFBP4	1.51 ± 0.33	4.00E-126
16	P07357	CO8A	Complement component C8 alpha chain	C8A	1.79 ± 0.23	0
17	O00187	MASP2	Mannan-binding lectin serine protease 2	MASP2	2.69 ± 0.98	0
18	P02749	APOH	Beta-2-glycoprotein 1	APOH	1.32 ± 0.26	2.00E-108
19	Q9NZP8	C1RL	Complement C1r subcomponent-like protein	C1RL	2.07 ± 0.18	0
20	P48740	MASP1	Mannan-binding lectin serine protease 1	MASP1	2.57 ± 0.99	0
21	P05164	PERM	Myeloperoxidase	MPO	2.24 ± 0.82	0
22	Q15485	FCN2	Ficolin-2	FCN2	2.52 ± 0.65	6.00E-158
23	P26927	HGFL	Hepatocyte growth factor-like protein	MST1	1.51 ± 0.42	0
24	P01876	IGHA1	Ig alpha-1 chain C region	DKFZp686K18196	1.85 ± 0.9	0
25	P02788	TRFL	Lactotransferrin	LTF	3.3 ± 1.16	0
26	P07360	CO8G	Complement component C8 gamma chain	C8G	1.4 ± 0.15	2.00E-103
27	P36980	FHR2	Complement factor H-related protein 2	CFHR2	4.84 ± 1.31	3.00E-137
28	P02743	SAMP	Serum amyloid P-component	APCS	2.1 ± 0.23	4.00E-129
29	Q03591	FHR1	Complement factor H-related protein 1	CFHR1	2.41 ± 0.55	0
30	Q99969	RARR2	Retinoic acid receptor responder protein 2	RARRES2	1.43 ± 0.25	4.00E-91
31	P01859	IGHG2	Ig gamma-2 chain C region	DKFZp686I04196	1.9 ± 0.92	2.00E-180
32	Q96KN2	CNDP1	Beta-Ala-His dipeptidase	CNDP1	1.36 ± 0.15	0
33	P01764	HV303	Ig heavy chain V-III region VH26	VH3	2.28 ± 1.04	4.00E-40
34	Q03591	FHR1	Complement factor H-related protein 1	FHR-1	3.66 ± 2.08	8.00E-15
35	O00602	FCN1	Ficolin-1	FCN1	2.81 ± 0.92	0
36	Q16769	QPCT	Glutaminyl-peptide cyclotransferase	QPCT	1.82 ± 0.73	0
37	P61628	LYSC	Lysozyme C	LYZ	3.61 ± 1.21	2.00E-82
38	P48740	MASP1	Mannan-binding lectin serine protease 1	DKFZp686M0562	1.88 ± 0.49	6.00E-51

**Table 3:** Down-regulated proteins, identified in aged of human

Number	Accession	protein symbol	Description	gene symbol	Mean ± SD	E_Value
1	Q76FE5	H2B	Histone H2B	ABCF2	0.31 ± 0.1	6.00E-28
2	Q13201	MMRN1	Multimerin-1	MMRN1	0.77 ± 0.18	0
3	P08571	CD14	Monocyte differentiation antigen CD14	CD14	0.48 ± 0.04	0
4	P13796	PLSL	Plastin-2	HEL-S-37	0.19 ± 0.08	0
5	P05556	ITB1	Integrin beta-1	ITGB1	0.42 ± 0.03	0
6	P02776	PLF4	Platelet factor 4	PF4	0.48 ± 0.17	2.00E-40
7	Q6Q788	APOA5	Apolipoprotein A-V	APOA5	0.75 ± 0.14	0
8	Q96PD5	PGRP2	N-acetylmuramoyl-L-alanine amidase	PGLYRP2	0.46 ± 0.14	0
9	P08697	A2AP	Alpha-2-antiplasmin	SERPINF2	0.51 ± 0.13	0
10	Q9UK55	ZPI	Protein Z-dependent protease inhibitor	SERPINA10	0.38 ± 0.1	0
11	P19823	ITIH2	Inter-alpha-trypsin inhibitor heavy chain H2	ITIH2	0.65 ± 0.08	0
12	P02760|	AMBP	Protein AMBP	AMBP	0.38 ± 0.09	0
13	Q96KK5	H2A1H	Histone H2A type 1-H	HIST1H2AG	0.49 ± 0.07	2.00E-54
14	P04275	VWF	von Willebrand factor	VWF	0.48 ± 0.08	0
15	P12111	CO6A3	Collagen alpha-3(VI) chain	COL6A3	0.43 ± 0.09	0
16	Q9UGM5	FETUB	Fetuin-B	FETUB	0.24 ± 0.04	0
17	P15169	CBPN	Carboxypeptidase N catalytic chain	CPN1	0.71 ± 0.12	0
18	P22792	CPN2	Carboxypeptidase N subunit 2	CPN2	0.7 ± 0.2	0
19	Q5XIF6	TBA4A	Tubulin alpha-4A chain	TUBA4A	0.83 ± 0.18	0
20	P29622	KAIN	Kallistatin	SERPINA4	0.2 ± 0.07	0
21	P05452	TETN	Tetranectin	CLEC3B	0.43 ± 0.02	4.00E-116
22	P35858	ALS	Insulin-like growth factor-binding protein complex acid labile subunit	IGFALS	0.58 ± 0.14	0
23	P04196	HRG	Histidine-rich glycoprotein	HRG	1.76 ± 0.63	0
24	Q13790	APOF	Apolipoprotein F	APOF	1.40 ± 0.43	0
25	P55058	PLTP	Phospholipid transfer protein	PLTP	1.39 ± 0.11	0
26	P04070	PROC	Vitamin K-dependent protein C	LOC	1.87 ± 0.64	2.00E-35
27	P01764	HV303	Ig heavy chain V-III region VH26	IGHV3–49	1.32 ± 0.24	5.00E-42
28	O95445	APOM	Apolipoprotein M	APOM	1.40 ± 0.25	2.00E-110
29	P06702	S10A9	Protein S100-A9	S100	3.10 ± 1.59	6.00E-62
30	Q5R1X3	ACTB	Actin, cytoplasmic 1	LOC	2.23 ± 0.62	0
31	P05543	THBG	Thyroxine-binding globulin	SERPINA7	2.26 ± 0.56	0
32	P04278	SHBG	Sex hormone-binding globulin	SHBG	3.77 ± 0.14	4.00E-171
33	P10720	PF4V	Platelet factor 4 variant	PF4V1	1.35 ± 0.31	6.00E-33
34	P00748	FA12	Coagulation factor XII	F12	1.69 ± 0.70	0
35	P36955	PEDF	Pigment epithelium-derived factor	SERPINF1	1.45 ± 0.26	0
36	P19827	ITIH1	Inter-alpha-trypsin inhibitor heavy chain H1	ITIH1	2.16 ± 0.16	0

### KEGG pathway analysis of differentially expressed proteins

**Fig. 3:**
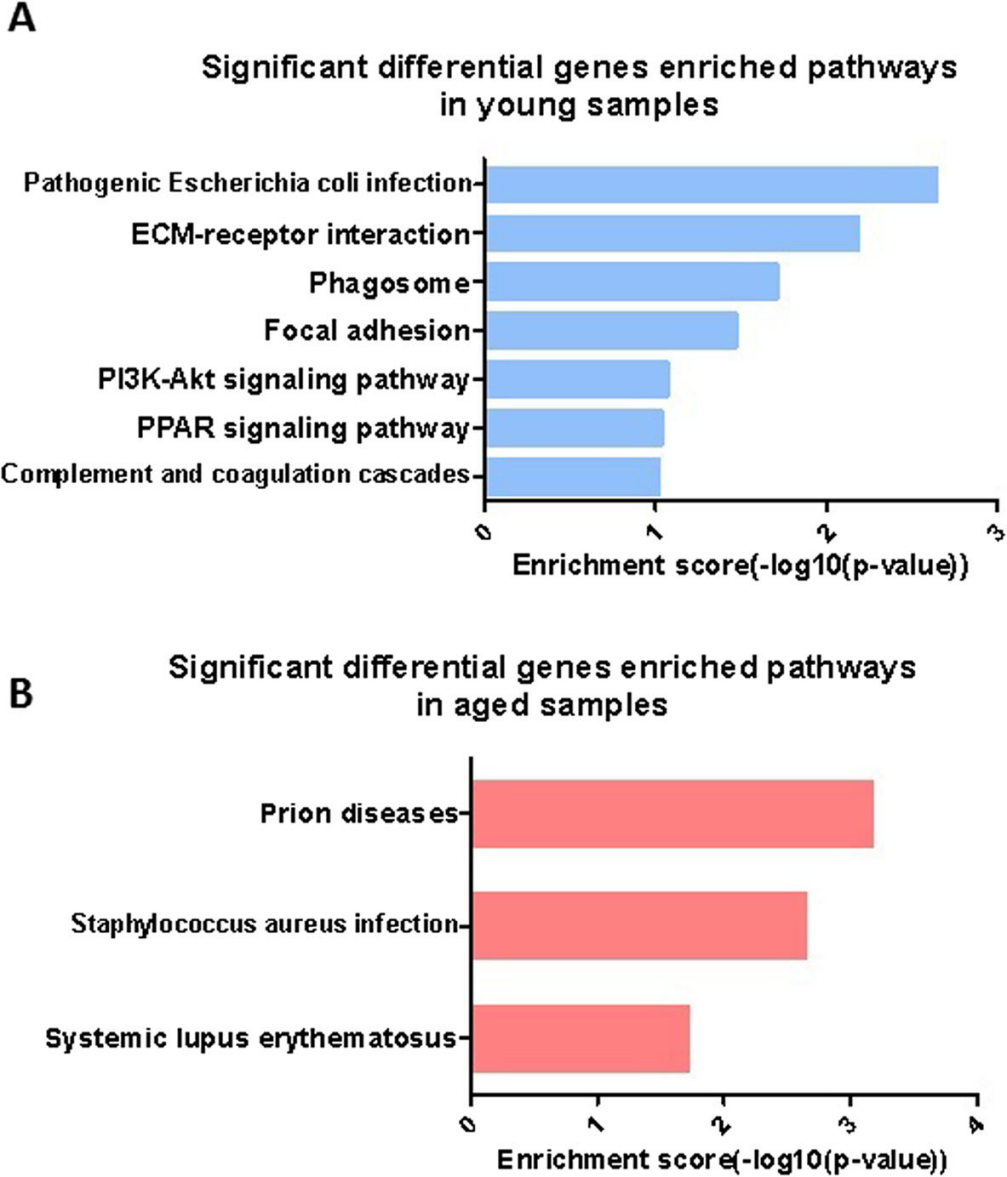
KEGG pathway analysis of differentially expressed proteins in human samples. (**a**) Significant differential genes enriched pathways in young samples. (**b**) Significant differential genes enriched pathways in aged samples

In human aged samples, differentially expressed plasma proteins were mainly enriched in three pathways, while in young plasma samples, differentially expressed plasma proteins were mainly enriched in seven pathways by KEGG pathway analysis. These included Pathogenic *Escherichia coli* infection, ECM-receptor interaction, Phagosome, Focal adhesion, PI3K-Akt signaling pathway and PPAR signaling pathway (Fig. 3).

### Validation of iTRAQ results in individual subjects

**Fig. 4:**
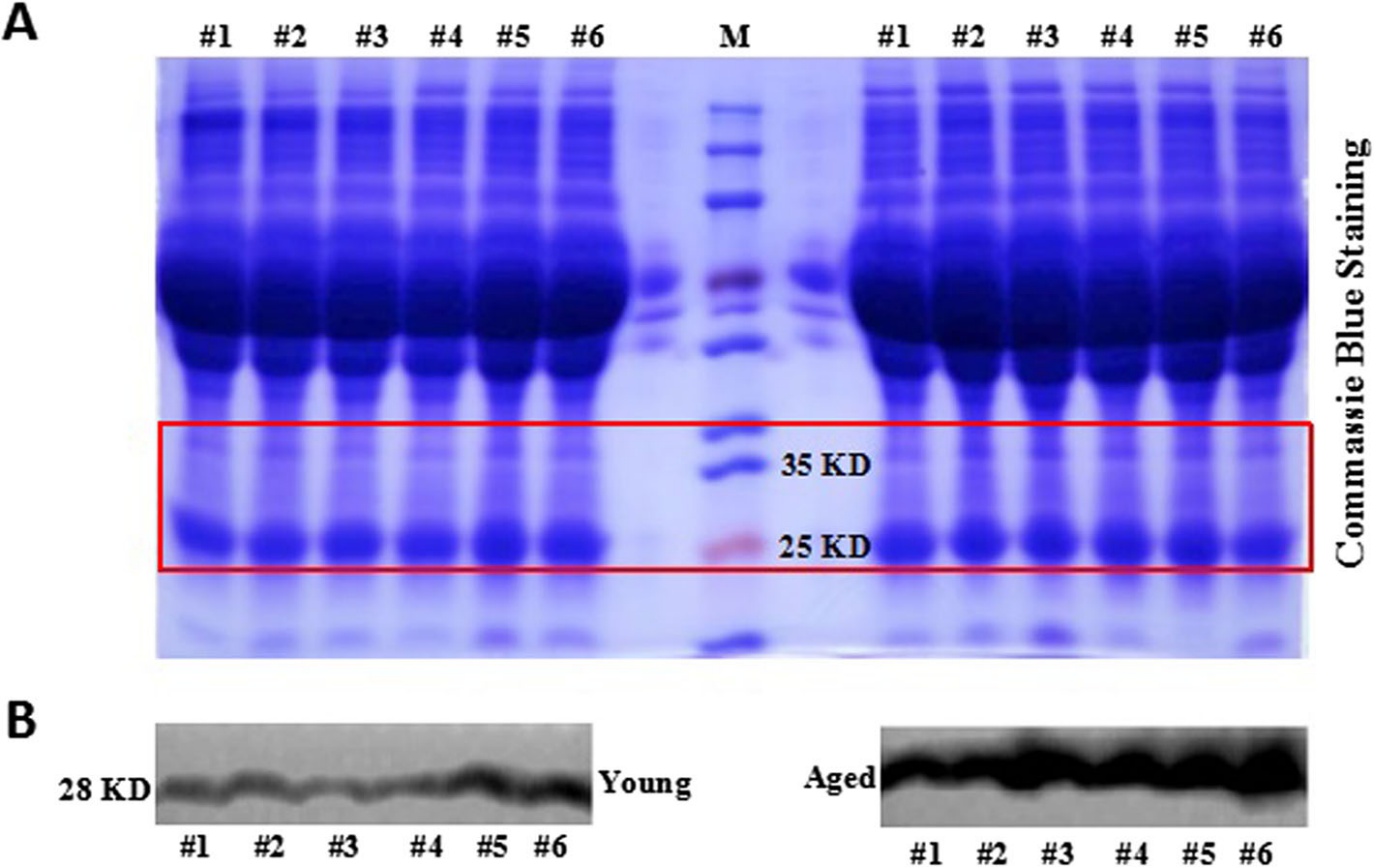
Expression levels of IGFBP4 revealed by western blotting. (**a**) Coomassie blue staining of total protein loading; (**b**) Western blot analysis of IGFBP4 in human young and aged plasma

To confirm the accuracy of our iTRAQ results, a key component that triggers senescence in young MSC [20], IGFBP4, was selected for further study. Consistent with the results of the data in the Table 2 and Additional file 3: Table S1, the protein level of IGFBP4 was significantly up-regulated in the aged group compared with the young group (Fig. 4).

### IGFBP4 impaired cognitive performance of mice

**Fig. 5:**
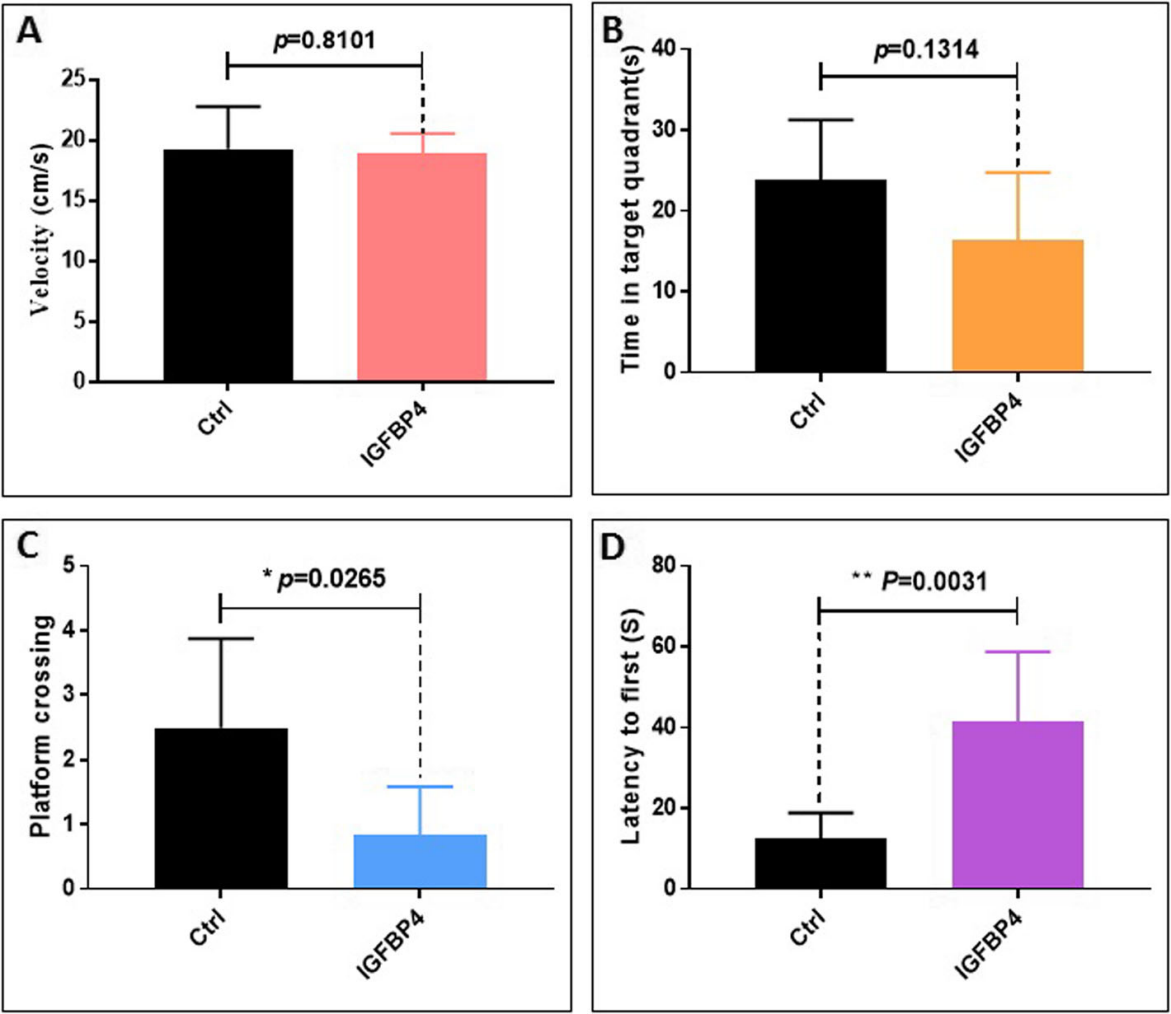
Morris water maze analysis of behavior of 8 months mice injected with IGFBP4 protein. (**a**) The swimming speed of the two groups of mice. (**b**) The time spent in the target quadrant of the two groups of mice (*p*=0.1314 ). (**c**) Number of times the two groups of mice crossed the platform (*p< 0.05). (**d**) The time of the two groups of mice first appeared on the platform (**p<0.01)

It is well recognized that aging can be accompanied by cognitive impairment. To evaluate the effects of IGFBP4 on cognitive performance, through tail vein injection at a does of 2.67 μg/kg, once every other day over the course of 1 month, into mice with 8 months ages, 13 mices were divided into two groups: Control group (*n* = 6) and IGFBP4 group (*n* = 7), we examined mice in the Morris water maze. During the test time, there was no significant difference between the two groups in swimming speed (Fig. 5a). Although there was no significant difference between the two groups, the duration time in the quadrant where the platform was previously placed that the IGFBP4 group spent was less than the control group (Fig. 5b). Meanwhile, compared with the control group, the mice of the IGFBP4 group also significantly reduced the number of times they crossed over the previous position of the platform (Fig. 5c). Moreover, the IGFBP4 group showed a significant increase in the time getting to the stage for the first time compared with the control group (Fig. 5d). These results demonstrated that IGFBP4 could aggravate the dysfunction of cognitive ability in mice.

## Discussions

Aging is a phenomenon that results from a complex interaction of diverse factors. The mechanisms underlying aging are very intricate and many factors such as aging cells and tissue remodeling, telomeres, DNA repair, growth, energy homeostasis, and reproductive function have been implicated in the process of aging [1]. Although many factors related to aging have been identified, the overall mechanism of aging is highly complicated, so specific causes of aging are still unclear. Proteins circulating in the blood are critical for aging; however, the serum proteome from human and monkey has remained largely unexplored. Therefore, studies at the proteome level would help us to better understand the changes in the mechanisms associated with aging.

In our study, 74 proteins and 69 proteins were identified differentially expressed in aged plasma compared with young plasma in human and monkey samples, respectively. Through GO annotation analysis, it was revealed that most of the significant proteins in aging belong to cell parts, extracellular region, and organelle, and mainly participate in the cellular process, metabolic process, and single-organism process. It was revealed that phagosome, complement and coagulation cascades, primary immunodeficiency, dilated cardiomyopathy, and viral myocarditis pathways are the main signaling pathways activated in aging via KEGG pathway analysis, indicating that cardiovascular diseases and inflammatory responses may play a significant role in the process of aging.

### Association between IGFBPs and aging

Insulin-like growth factor I (IGFI) and IGFII are members of the insulin superfamily of growth-promoting peptides and are among the most abundant and ubiquitous polypeptide growth factors. The IGFs are distinguished from insulin by their interaction in circulating blood and the cellular environment with six high-affinity IGF binding proteins (IGFBPs) that have multiple functional properties [21]. In addition to altering the equilibrium between the IGFs and their cell-surface receptors, the IGFBPs can also regulate signaling what is stimulated by other growth factors through their distinct receptors [21].

Almost all members of IGFBP family, such as IGFBP1/2/3/4/5, are involved in coordinately regulating growth within the physiological context of changing metabolic demands [22]. Several studies have identified that IGFBPs can affect biological responses by interacting with cell-surface proteins [22]. IGFBP1 and 2 can modulate cell migration and adhesion by binding to α5β1 integrin [23, 24]. IGFBP2 induces activation of the PI3 kinase/AKT pathway through indirect action by binding to a cell-surface receptor termed receptor tyrosine phosphatase beta (RPTPβ) [25, 26]. In addition, IGFBPs are involved in insulin resistance, lipid metabolism, diabetes,carbohydrate metabolism, bone metabolism, and atherosclerosis [22, 27–30].

As a member of IGFBP family, IGFBP4 mainly mediates the cellular signaling by banding to IGF1 that can limit the amount of free IGF in extracellular fluid that is available for receptor activation [22]. IGFBP4 can act as an inhibitor of the canonical Wnt signaling required for cardiogenesis and provides a molecular link between IGF signaling and Wnt signaling [31]. There is evidence that IGFBP4 expression increases with age and is associated with the impairment of MSC differentiation via the Erk and Smad pathway [32]. Consistent with previous studies that indicated IGFBP4 as a key component needed for triggering senescence in young MSC and a harmful factor that impair the habituation learning, which is correlated with cognitive function [20, 33], our present study demonstrated that IGFBP4 was up-regulated in the plasma of aged compared with young subjects and that injection of IGFBP4 into mice also accelerates cognitive dysfunction. IGFBP4 also plays a critical role in adipogenesis and loss of IGFBP4 can induce a decrease in adipose tissue [34].

### Association between proteolytic systems and aging

There is growing evidence that normal and pathological aging are often associated with impaired protein homeostasis or proteostasis [35]. Both mechanisms are involved in restoring the structure of misfolded polypeptides and in their removal and degradation, thus preventing the accumulation of damaged components and ensuring the continuous renewal of intracellular proteins [36, 37]. The function of these systems plays a vital role in the survival of cells. Additionally, the accumulation of unfolded, misfolded, or non-degraded proteins is a major factor in the development of some age-related pathologies, such as Alzheimer’s disease, Parkinson’s disease, and Huntington’s disease [38]. Moreover, it has been demonstrated that the activities of the ubiquitin-proteasome system and the autophagy-lysosomal system, which are the two principal proteolytic systems implicated in protein quality control, decrease with aging [38, 39], supporting the view that impaired proteostasis constitutes a common feature of old age.

Regarding autophagy, a double transgenic mouse model in which the amount of the lysosomal receptor for chaperone-mediated autophagy (CMA, one type of autophagy), does not result in an age-dependent decrease in autophagic activity and preserves improved hepatic function with aging [40]. Also, rapamycin, which is an inhibitor of the mTOR pathway, can induce macroautophagy (another form of autophagy different from CMA) to extend median and maximal lifespan of mice [41, 42]. Notably, it had been reported that rapamycin delays multiple aspects of aging in mice [43].

Consistent with the relevance of proteolytic systems in the process of aging, our present study also revealed that the Phagosome pathway plays a key role in the biology of aging. However, although its influence is not obvious in our study, another pathway, Fc gamma receptor-mediated phagocytosis, is also involved in the process. Therefore, all of the above indicate that aging is involved in perturbed proteostasis, and that experimental perturbation of proteostasis can precipitate age-associated pathologies [1].

### Association between inflammation and aging

In recent years a lot of previous researches found that aging also involves changes at the level of endocrine, neuroendocrine or neuronal intercellular communication [44–47]. Consistent with these results, our present study revealed involvement of Complement and coagulation cascades and Primary immunodeficiency pathway in aging at the protein level. Although the exact mechanism underling aging is still unknown, a prominent aging-associated alteration in intercellular communication, termed “inflammaging” has been widely accepted as a hallmark associated with aging [48, 49]. Inflammaging may result from multiple causes, such as genetic susceptibility, visceral obesity, microbiota and gut permeability, cellular senescence, impaired recycling and elimination of degraded cellular material, or intrinsic defects in immune cells and chronic infections [8]. Inflammation is also associated with the pathogenesis of obesity and type 2 diabetes, two conditions that contribute to and correlate with aging in the human population [50]. Similarly, defective inflammatory responses play a critical role in atherosclerosis [51]. At the same time, modulating inflammaging as a promising strategy to slow the decline of health that occurs with aging has become more and more widely accepted [8]. Thus, it is reasonable to conclude that pathways related to inflammation play a crucial role in the biology of aging.

## Conclusions

In summary, due to the extremely large dynamic range of plasma proteins and some inherent defects of the iTRAQ [52, 53], we did not identify many specific proteins related to aging compered with other subjects [52, 54]. Our results yet revealed that the differentially expressed IGFBP4 between aged and young samples is related to the process of aging. In addition,our present study indicated that proteolytic systems and inflammatory responses may play a significant role in the process of aging. Thus, our study suggested a possible relationship between some growth factors, such as IGFBPs, proteolytic systems, and inflammatory responses can affect the process of body aging.. This may provide novel insight into the prevention of aging.

## Methods

### Animals

The mice used in the experiment were all C57BL/6, purchased from Beijing Vital River Laboratory Animal Technology Co., Ltd. Mice were maintained on a 12 h light/dark schedule, and allowed free access to food and water, following protocols approved by the Animal Research Committee of Tongji Hospital, Tongji University School of Medicine, China. After the test of Morris water maze, the mice were anesthetized with tribromoethanol (Sigma, Saint Louis, USA), then the mice were killed by cervical dislocation.

### Sample collection and protein extraction

For LC-ESI-MS/MS, plasma samples from eight humans and eight *Macaca fascicularis* monkeys were collected and divided into four groups: Aged-Human (H), Young-H, Aged-Monkey (M) and Young-M. The plasma sample from each individual was stored at − 80 °C for further testing.

To reduce the complexity of samples, the highly abundant proteins were depleted using ProteoMinerTM Kits (Bio-Rad Laboratories, Hercules, CA, USA) according to the manufacturer’s protocol. The smaples was added to a mixture of protein lysate, 1 mM PMSF, and 2 mM EDTA. 5 minters later, adding 10 mM DTT. After 15 min of ultrasonic lysis, then centrifuged at 25,000×g for 20 min. The supernatant was added 5 times volume of precooled acetone and precipitated at − 20 °C for 2 h, then centrifuged for 20 min with 16,000×g. Follow the previous steps for another precipitation cracking. Then the supernatant was treated with 10 mM DTT for 1 h at 56 °C, followed by adding 55 mM IAM in a dark room for 45 min. Next, appropriate amount of cold acetone was add to the sample, incubating at − 20 °C for 2 h. After centrifugation of 25,000×g for 20 min, the precipitation was dissolved in 200 uL 0.5 M TEAB for 15 min of ultrasonic lysis, followed by another centrifugation of 25,000×g for 20 min. The protein concentration of each sample was determined by using the BCA Kit (Sango Bioteh, Shanghai, China).

### iTRAQ labeling

Proteins (100 μg) were digested with trypsin (protein:trypsin = 20:1; Sigma) and incubated at 37 °C for 4 h. More trypsin was then added at the above proportion and incubation continued at 37 °C for 8 h. The peptides were then dried with a vacuum centrifugal pump and 0.5 M TEAB was used to resolubilize the peptides. Peptides were then labeled with iTRAQ with incubation at room temperature for 2 h according to the manufacturer’s instructions. The labeled peptides were mixed and separated on an SCX column through the LC-20AB liquid phase system (Shimadzu, Kyoto, JPN).

### LC-ESI-MS/MS analysis based on TripleTOF 5600

Each component was redissolved to about 0.5 μg/μl in buffer A (5% ACN, 0.1% FA) and centrifuged for 10 min to remove insoluble matter. Five-microliter samples of each component (about 2.5 μg protein) were separated by the LC-20 AD system (Shimadzu, Kyoto Japan). The separation procedure was as follows: first, the sample was transferred to the Trap column over 4 min at a flow rate of 8 μL/min. An analytical gradient with a total flow rate of 300 μL/min was then added to bring the sample into the column. Then the sample was separated and transferred to the mass spectrometry system. Elution was performed under 5% buffer B (95% ACN, 0.1% FA) for 5 min, followed by a linear gradient for 35 min in which the proportion of buffer B increased from 5 to 35%. The proportion was then increased to 60% for the next 5 min, then increased to 80% over 2 min where it remained for 2 min. Finally the proportion of buffer B was recovered to 5% over 1 min and balanced for 10 min. The machine used was a TripleTOF 5600 (AB SCIEX, Concord, ON) with a Nanospray IIIsource (AB SCIEX, Concord, ON) ion source and a quartz radiator (New Objectives, Woburn, MA).

### Bioinformatics analysis

The database was used in this study: Uniprot_homo (127497sequences), link address: (http://www.uniprot.org/uniprot/?query=taxonomy:9606). The Software Mascot was used to identify protein, version Mascot2.3.02. It has been rated as the gold standard of biological mass spectrometry software by Frost/Sullivan research institutions. Gene Ontology (GO) database (http://www.geneontology.org.) was used to classified the functional annotation of proteins into 3 categories: biological process, molecular function, and cellular component. The significant enrichment analysis of GO function was performed to identify GO terms that were significantly enriched in differentially expressed proteins. When the protein abundance ratio was more than 1.5 times and its *p*-value was less than 0.05, the protein was regarded as the differential protein between different samples. The significant enrichment analysis of Pathway is based on KEGG Pathway to find out the significant enrichment of Pathway in differential proteins compared with all the identified protein backgrounds.

### Western blotting

To validate the accuracy of iTRAQ results, western blot analysis was performed.

The plasma was diluted ten times by PBS. 30 μL of samples per lane were loaded for 10% Bis-Tris polyacrylamide gels and then transferred onto PVDF membrane (Millipore, Billerica, MA) by electroblotting. The membranes were blocked in TBS 0.5% Tween containing 5% skim milk powder (OXOID, Basingstoke, UK) for 1 h at room temperature and incubated with primary antibody: anti-IGFBP4 (Abcam, Cambridge, UK) overnight at 4 °C. Then the membranes were incubated with peroxidase-conjugated secondary antibodie (Bio-Rad, Hercules, USA) for 1 h at room temperature. The bands were visualized by using Millipore’s enhanced chemiluminescence (ECL) with the Amersham Imager 600 detection system (GE, Boston, USA). The western blot results represent at least 2 independent biological replicates.

### Behavioral test

Spatial learning and cognitive flexibility were evaluated using a Morris water maze (WMW) [55, 56], which consisted of a circular pool (150 cm in diameter), filling with water maintained at room temperature (23 ± 1 °C), opacified by adding nontoxic white. The platform (diameter in 15 cm) was hidden 1 cm underneath the water surface in the middle of one fixed quadrant (‘target’) of the pool. Four different colors and dimensions visual cues were attached to the side of the pool equidistant from one another, and the pool was surrounded by a plain curtain to block any other visual cues. For training (day 1–5), mice were released randomly with their heads facing the pool wall from one of the four starting locations, and trained daily in four sequential sessions (15–30 min interval). Once mice were not able to locate the platform after 60 s, they were manually placed on the platform and allowed to remain on it for 20 s. For probe test (day 6), a 60 s probe trial was performed to assess the memory of the mice. The latency to locate the platform and velocity were recorded by EthoVision video tracking equipment and software (Noldus, Wageningen, The Netherlands).

### Statistical analysis

The data are reported as the means ± SD, and statistical analysis between two groups was assessed using independent-samples t-tests or Mann-Whitney U tests. *p* < 0.05 was considered statistically significant. All the statistical analyses were performed using SPSS software, version 22 (IBM, Armonk, USA) and GraphPad Prism, version 7 (GraphPad Software Inc.,San Diego, USA).

## Supplementary information

**Additional file 1: Figure S1.** Extended monkey plasma proteome dataset from iTRAQ shotgun analysis.

**Additional file 2: Figure S2.** Gene ontology (GO) annotation and functional classification of identified plasma proteins from all monkey samples. GO terms for cellular compartment (A), molecular function (B), and biological process (C).

**Additional file 3: Table S1.** Up-regulated proteins in aging of monkey.

**Additional file 4: Table S2.** Down-regulated proteins in aging of monkey.

## Data Availability

The datasets used and/or analysed during the current study available from the corresponding author on reasonable request.

## Abbreviations

DAVID: Database for Annotation, Visualization and Integrated Discovery2
GO: Gene Ontology
iTRAQ: isobaric tags for relative and absolute quantification
TNF-α: tumor necrosis factor α
