# Supplementary figures and images for: Quantitative iTRAQ-based proteomic analysis of differentially expressed proteins in aging in human and monkey
Source: BMC Genomics. 2019 Oct 11;20:725. doi: 10.1186/s12864-019-6089-z (PMC6788010; doi:10.1186/s12864-019-6089-z)

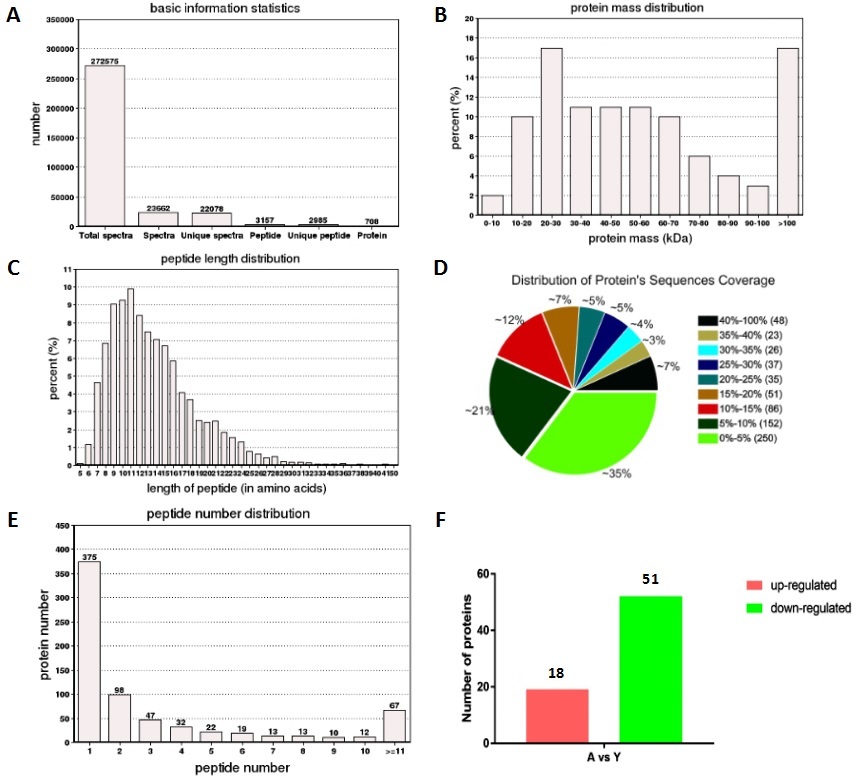

Supplement: Supplementary file 1 — Additional file 1: Figure S1. Extended monkey plasma proteome dataset from iTRAQ shotgun analysis. [file 12864_2019_6089_MOESM1_ESM.jpg]

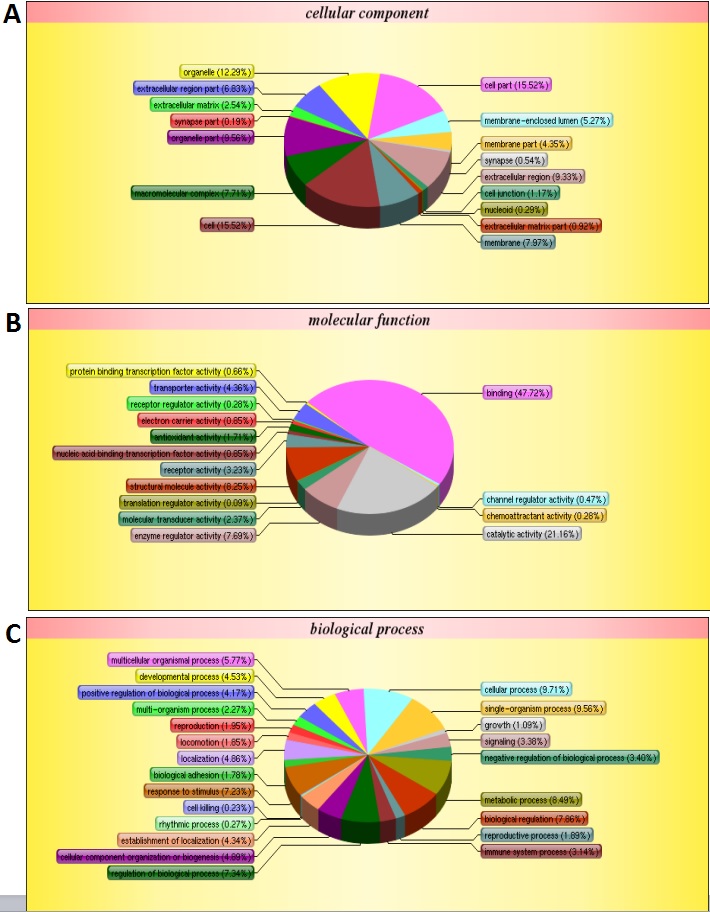

Supplement: Supplementary file 2 — Additional file 2: Figure S2. Gene ontology (GO) annotation and functional classification of identified plasma proteins from all monkey samples. GO terms for cellular compartment (A), molecular function (B), and biological process (C). [file 12864_2019_6089_MOESM2_ESM.jpg]
