# Supplementary material for: Quantitative iTRAQ-based proteomic analysis of differentially expressed proteins in aging in human and monkey
Source: BMC Genomics. 2019 Oct 11;20:725. doi: 10.1186/s12864-019-6089-z (PMC6788010; doi:10.1186/s12864-019-6089-z)
Supplement: Supplementary file 3 — Additional file 3: Table S1. Up-regulated proteins in aging of monkey. [file 12864_2019_6089_MOESM3_ESM.doc]

Supplementary **table 1**. Up-regulated proteins in aging of monkey

| **Accession** | **protein symbol** | **Description** | [**Mean±SD**](mailto:A-VS-Y@Mean±SD) | **E_Value** |
| --- | --- | --- | --- | --- |
| Q5RD26 | HNRH2 | Heterogeneous nuclear ribonucleoprotein H2 | 1.89±0.43 | 0 |
| Q62812 | MYH9 | Myosin-9 | 1.38±0.3 | 1.00E-107 |
| P01857 | IGHG1 | Ig gamma-1 chain C region | 1.5±0.63 | 3.00E-155 |
| Q95140 | RLA0 | 60S acidic ribosomal protein P0 | 3.97±1.64 | 1.00E-150 |
| Q4R4N7 | GANAB | Neutral alpha-glucosidase AB | 4.8±1.81 | 0 |
| Q8TED1 | GPX8 | Probable glutathione peroxidase 8 | 9.32±6.15 | 4.00E-109 |
| Q02985 | FHR3 | Complement factor H-related protein 3 | 3.02±2.12 | 3.00E-66 |
| P02738 | SAA | Amyloid protein A | 2.89±1.3 | 2.00E-37 |
| Q8WN63 | ANGI | Angiogenin | 1.6±0.13 | 1.00E-73 |
| P11142 | HSP7C | Heat shock cognate 71 kDa protein | 2.95±0.24 | 0 |
| Q9Y4L1 | HYOU1 | Hypoxia up-regulated protein 1 | 1.8±0.23 | 0 |
| Q09666 | AHNK | Neuroblast differentiation-associated protein AHNAK | 3.63±1.49 | 0 |
| P08603 | CFAH | Complement factor H | 2.44±0.75 | 2.00E-153 |
| Q1KLX7 | CAMP | Cathelicidin antimicrobial peptide | 1.61±0.22 | 7.00E-81 |
| P22692 | IGFBP4 | Insulin-like growth factor-binding protein 4 | 1.318±0.2156 | 4.00E-126 |
| P05109 | S10A8 | Protein S100-A8 | 2.1±1.1 | 3.00E-44 |
| Q9NR56 | MBNL1 | Muscleblind-like protein 1 | 6.87±2.12 | 6.00E-179 |
| P69893 | TBB5 | Tubulin beta-5 chain | 1.69±0.58 | 0 |
| P06702 | S10A9 | Protein S100-A9 | 2.06±0.95 | 1.00E-47 |
| Q8HZQ0 | RNAS4 | Ribonuclease 4 | 2±0.62 | 4.00E-73 |
| Q92743 | HTRA1 | Serine protease HTRA1 | 1.31±0.39 | 0 |
| P05386 | RLA1 | 60S acidic ribosomal protein P1 | 2.48±0.64 | 4.00E-18 |
| Q2TBQ5 | RL7A | 60S ribosomal protein L7a | 336.8±574.3 | 9.00E-128 |
| Q14520 | HABP2 | Hyaluronan-binding protein 2 | 1.5±0.24 | 0 |
| Q96PD5 | PGRP2 | N-acetylmuramoyl-L-alanine amidase | 1.61±0.44 | 0 |
| P62082 | RS7 | 40S ribosomal protein S7 | 3.93±2.38 | 2.00E-107 |
| P02763 | A1AG1 | Alpha-1-acid glycoprotein 1 | 2.05±0.93 | 2.00E-91 |
| P02653 | APOA2 | Apolipoprotein A-II | 1.47±0.21 | 6.00E-38 |
| Q7Z406 | MYH14 | Myosin-14 | 1.41±0.27 | 0 |
| Q08830 | FGL1 | Fibrinogen-like protein 1 | 2.42±1.97 | 4.00E-86 |
| P63261 | ACTG | Actin, cytoplasmic 2 | 4.57±3.02 | 4.00E-112 |
| P04163 | S10AA | Protein S100-A10 | 10.57±6.51 | 4.00E-51 |
| P35579 | MYH9 | Myosin-9 | 1.54±0.27 | 0 |
| P79324 | RL15 | 60S ribosomal protein L15 | 30.78±31.16 | 2.00E-71 |
| P39023 | RL3 | 60S ribosomal protein L3 | 12.71±3.41 | 2.00E-57 |
| P02533 | K1C14 | Keratin, type I cytoskeletal 14 | 1.45±0.33 | 0 |
| P06576 | ATPB | ATP synthase subunit beta, mitochondrial | 2.77±0.39 | 0 |
| P61585 | RHOA | Transforming protein RhoA | 8.43±2.96 | 5.00E-62 |
| O88569 | ROA2 | Heterogeneous nuclear ribonucleoproteins A2/B1 | 6.79±1.65 | 9.00E-105 |
| P13645 | K1C10 | Keratin, type I cytoskeletal 10 | 1.57±0.89 | 0 |
| Q95LB0 | APOH | Beta-2-glycoprotein 1 | 1.74±0.83 | 2.00E-146 |
| P62849 | RS24 | 40S ribosomal protein S24 | 16.03±8.17 | 2.00E-52 |
| Q9Y490 | TLN1 | Talin-1 | 1.47±0.33 | 0 |
| Q9MZA9 | VIME | Vimentin | 3.26±0.62 | 7.00E-62 |
| P48740 | MASP1 | Mannan-binding lectin serine protease 1 | 1.89±1.37 | 2.00E-109 |
| P12235 | ADT1 | ADP/ATP translocase 1 | 4.43±1.38 | 2.00E-166 |
| Q08758 | LCAT | Phosphatidylcholine-sterol acyltransferase | 1.42±0.42 | 3.00E-21 |
| Q5RBP8 | PROP | Properdin | 1.97±1.02 | 0 |
| P36955 | PEDF | Pigment epithelium-derived factor | 1.76±0.36 | 5.00E-113 |
| Q5R4R8 | EF1A1 | Elongation factor 1-alpha 1 | 335.7±575.3 | 0 |
| P02545 | LMNA | Prelamin-A/C | 3.95±1.35 | 0 |
