# Supplementary material for: Quantitative iTRAQ-based proteomic analysis of differentially expressed proteins in aging in human and monkey
Source: BMC Genomics. 2019 Oct 11;20:725. doi: 10.1186/s12864-019-6089-z (PMC6788010; doi:10.1186/s12864-019-6089-z)
Supplement: Supplementary file 4 — Additional file 4: Table S2. Down-regulated proteins in aging of monkey. [file 12864_2019_6089_MOESM4_ESM.doc]

Supplementary **table 2**. Down-regulated proteins in aging of monkey

| **Accession** | **Protein symbol** | **Description** | [**Mean±SD**](mailto:A-VS-Y@Mean±SD) | **E_Value** |
| --- | --- | --- | --- | --- |
| Q5R5A4 | CFAI | Complement factor I | 0.49±0.2 | 2.00E-150 |
| P0C0L4 | CO4A | Complement C4-A | 0.65±0.23 | 4.00E-148 |
| P01764 | HV303 | Ig heavy chain V-III region VH26 | 0.58±0.12 | 6.00E-49 |
| Q6ZMU1 | C3P1 | Putative protein C3P1 | 0.64±0.21 | 1.00E-30 |
| P02788 | TRFL | Lactotransferrin | 0.68±0.24 | 0 |
| Q99983 | OMD | Osteomodulin | 0.54±0.12 | 0 |
| O00748 | EST2 | Cocaine esterase | 0.63±0.47 | 0 |
| P04275 | VWF | von Willebrand factor | 0.63±0.21 | 0 |
| P05156 | CFAI | Complement factor I | 0.56±0.09 | 2.00E-28 |
| Q9Y5C1 | ANGL3 | Angiopoietin-related 3 | 0.64±0.02 | 0 |
| P27169 | PON1 | Serum paraoxonase/arylesterase 1 | 0.7±0.21 | 0 |
| P01042 | KNG1 | Kininogen-1 | 0.7±0.18 | 0 |
| P02776 | PLF4 | Platelet factor 4 | 0.37±0.18 | 6.00E-40 |
| P0CF74 | LAC6 | Ig lambda-6 chain C region | 0.75±0.25 | 3.00E-51 |
| Q9UGM5 | FETUB | Fetuin-B | 0.72±0.25 | 0 |
| Q8TF66 | LRC15 | Leucine-rich repeat-containing protein 15 | 0.63±0.13 | 0 |
| P05160 | F13B | Coagulation factor XIII B chain | 0.67±0.06 | 0 |
| Q5R5A4 | CFAI | Complement factor I | 0.4±0.05 | 3.00E-59 |
| P0CF74 | LAC6 | Ig lambda-6 chain C region | 0.56±0.3 | 1.00E-50 |
| P01614 | KV201 | Ig kappa chain V-II region Cum | 0.51±0.03 | 2.00E-46 |
